# Co-expression analysis of pancreatic cancer proteome reveals biology and prognostic biomarkers

**DOI:** 10.1007/s13402-020-00548-y

**Published:** 2020-08-29

**Authors:** G. Mantini, A. M. Vallés, T. Y. S. Le Large, M. Capula, N. Funel, T. V. Pham, S. R. Piersma, G. Kazemier, M. F. Bijlsma, E. Giovannetti, C. R. Jimenez

**Affiliations:** 1 Amsterdam UMC, Vrije Universiteit Amsterdam, Department of Medical Oncology, Cancer Center Amsterdam, Amsterdam, The Netherlands; 2 Fondazione Pisana Per La Scienza, Pisa, Italy; 3 Amsterdam UMC, Univ of Amsterdam, Laboratory for Experimental Oncology and Radiobiology, Amsterdam, The Netherlands; 4 Amsterdam UMC, Vrije Universiteit Amsterdam, Department of Surgery, Amsterdam, The Netherlands; 5 U.O. Anatomia ed Istologia Patologica II Azienda Ospedaliero Universitaria Pisana , Pisa, Italy; 6 Oncode Institute, Amsterdam, The Netherlands

**Keywords:** Pancreatic cancer, Protein co-expression, Systems biology, Proteomics, WGCNA, Prognostic biomarkers

## Abstract

**Purpose:**

Despite extensive biological and clinical studies, including comprehensive genomic and transcriptomic profiling efforts, pancreatic ductal adenocarcinoma (PDAC) remains a devastating disease, with a poor survival and limited therapeutic options. The goal of this study was to assess co-expressed PDAC proteins and their associations with biological pathways and clinical parameters.

**Methods:**

Correlation network analysis is emerging as a powerful approach to infer tumor biology from omics data and to prioritize candidate genes as biomarkers or drug targets. In this study, we applied a weighted gene co-expression network analysis (WGCNA) to the proteome of 20 surgically resected PDAC specimens (PXD015744) and confirmed its clinical value in 82 independent primary cases.

**Results:**

Using WGCNA, we obtained twelve co-expressed clusters with a distinct biology. Notably, we found that one module enriched for metabolic processes and epithelial-mesenchymal-transition (EMT) was significantly associated with overall survival (*p* = 0.01) and disease-free survival (*p* = 0.03). The prognostic value of three proteins (SPTBN1, KHSRP and PYGL) belonging to this module was confirmed using immunohistochemistry in a cohort of 82 independent resected patients. Risk score evaluation of the prognostic signature confirmed its association with overall survival in multivariate analyses. Finally, immunofluorescence analysis confirmed co-expression of SPTBN1 and KHSRP in Hs766t PDAC cells.

**Conclusions:**

Our WGCNA analysis revealed a PDAC module enriched for metabolic and EMT-associated processes. In addition, we found that three of the proteins involved were associated with PDAC survival.

**Electronic supplementary material:**

The online version of this article (10.1007/s13402-020-00548-y) contains supplementary material, which is available to authorized users.

## Introduction

Pancreatic ductal adenocarcinoma (PDAC) is the most common tumor type of the pancreas with a five-year survival rate not exceeding 8% [1]. A lack of reliable markers for early diagnosis, as well as its aggressive metastatic spread are the main causes of this extremely poor survival rate [2, 3]. The development of next-generation sequencing (NGS) has enabled detailed analyses of genomic aberrations and dysregulated gene expression patterns that underlie tumor development and progression, with KRAS, TP53, CDKN2A and SMAD4 as major oncogenic drivers of this disease. As yet, a comprehensive proteomic analysis of clinical PDAC samples is missing.

In recent years, multiple statistical methods and freely available bioinformatics tools have been developed that can extrapolate important features from high-throughput data, e.g. pinpointing genes associated with clinical parameters such as cancer status or patient survival [4]. In this context, networks based on co-expression data [5] have extensively been used to identify densely interconnected genes associated with phenotypic traits. Most of the available algorithms have been applied to microarray- and RNAseq-based expression data [6, 7]. Using these approaches Tang et al. [8], for example, identified new prognostic markers in breast cancer. Additionally, these approaches have been used to search for potential therapeutic targets in small-cell lung carcinoma [9]. More recently, an integrative analysis of co-expression networks from proteomics and transcriptomics data in Alzheimer’s disease revealed protein-specific networks in both asymptomatic and symptomatic patients [10, 11].

Weighted gene co-expression network analysis (WGCNA) assumes that the strength of node-to-node connections is best quantified by measures derived from their correlations. In co-expression networks for biological data, we refer to nodes as “genes” or “proteins”. A glossary of network-related terms is reported in Table 1. Constructing co-expression networks is an effective way to characterize correlation patterns among nodes and to infer new biological functions of densely interconnected nodes called “modules”. Modules can be related to external sample traits such as patient survival, recurrence and disease/health state, in order to discover biomarkers or therapeutic targets. Such modules are indicated by the Module Eigenprotein (ME; with a size of 1 × 20 in the current cohort, this is the most representative vector of values for that module) that can be related to external sample traits. In summary, the goals of a WGCNA analysis are: (i) establishment of real associations between proteins (instead of associations based on previous findings), (ii) identification of pathways specific for the dataset under analysis, (iii) association of modules to external information that provide biologically meaningful modules and (iv) identification of key drivers in relevant modules that may serve as candidate biomarkers and/or therapeutic targets.

**Table 1:** Glossary of network-related terms

Term	Definition	Reference
scale-free topology	Description of a cellular network structure in a graph theory concept	[20]
co-expression network	The edges are determined by the pairwise correlations between two protein expression profiles.	[6]
module	Module is a cluster of highly interconnected proteins.	[6]
connectivity	In co-expression networks, the connectivity measures how correlated a protein is with all other network proteins.	[6]
static tree cut	The branches of the hierachical clustering are cut at the same height. This is the most simple procedure for module identification.	[21]
dynamic tree cut	The module are defined by a non-constant cut off on the hierarchical clustering branches. This approach starts from a static tree cut and iteratively combine or remove proteins from one module to the other one. The iteration stops only when the modules reach stability.	[21]
adjacency matrix	Matrix containing pairwise correlations raised to the power β of all proteins.	[6]
weighted co-expression network	The edges of a network are described by weights. In the study, the weight is the correlation between two proteins raisd to the power β. This is essential to enhance strong correlations and avoid random noise.	[6]
unweighted co-expression network	Network that solely inform you if two proteins are connected or not	[6]
signed co-expression network	The edges of the network provide the sign of correlation (positive or negative)	[6]
unsigned co-expression network	The edges of the network do not provide the sign of correlation	[6]
direct network	The edges of this network described the action of one protein to another one (e.g. protein A is a kinase that phosphorylates protein B). It gives the direction of the action.	[6]
undirect network	The direction of the action is unknown.	[6]
module eigenprotein	The module eigenprotein ME is a vector with the most representative values of the given module and corresponds to the first principal component of that module.	[6]
hub gene	This term is used as an abbreviation of “highly connected gene” or specifically in this study, highly connected protein.	[6]

Explanatory table of the main network-related terms including references

Cancer proteomics aims to uncover the molecular basis of this devastating disease and to elucidate associated pathway features that cannot be detected by transcriptomics analyses. In this study, we report a PDAC proteomics analysis based on mass spectrometry (MS) data coupled to WGCNA to define networks of highly correlated proteins with specific functions associated with patient prognosis. We show that one module strongly features metabolic pathways and mesenchymal (EMT) signatures. This co-expression module was found to be significantly associated with disease-free survival (DFS) and overall survival (OS). The prognostic value of three key proteins in this module was validated in an independent cohort of 82 patients. These three proteins individually or in combination were able to predict patient survival.

## Material and methods

### Patient samples

Approval from the Local Medical Ethical committee at the VU University Medical Center was received (#14038). All patients provided informed consent for tissue sampling, clinical data analysis, and molecular analysis. Snap-frozen tumor samples from 39 patients included between January 2014 until November 2015 were evaluated by the Department of Pathology (Amsterdam UMC, Amsterdam). After pathological revision, 20 samples were eligible for further analysis. A minimum of 5–10% tumor surface was needed for further processing in this study. Clinical parameters were collected prospectively, OS and DFS data were obtained from electronic patient records. One patient was censored for OS analysis, since this patient succumbed to complications after surgery, defined as mortality within 60 days after surgery.

### Protein isolation from bulk tumor tissue and sample preparation for mass spectrometry

Protein isolation was performed as previously described [12]. Briefly, protein lysates were separated on pre-cast 4%–12% gradient gels using the NuPAGE SDS-PAGE system (Invitrogen, Carlsbad, CA, USA). Gels were fixed in 50% ethanol/3% phosphoric acid solution, stained with Coomassie brilliant blue G-250 and then washed and dehydrated in 50 mM ammonium bicarbonate (ABC) once and 50 mM ABC/50% acetonitrile (ACN) twice. Gel lanes were cut into five bands, with each band sliced further into approximately 1 mm^3^ cubes. The gel cubes were washed and dehydrated once in 50 mM ABC and twice in 50 mM ABC/50% ACN. Subsequently, the gel cubes were reduced in 10 mM DTT/50 mM ABC at 56 °C for 1 h, after which the supernatants were removed and the gel cubes were alkylated in 50 mM iodoacetamide/50 mM ABC for 45 min at room temperature in the dark. Next, the gel cubes were washed with 50 mM ABC/50% ACN, dried in a vacuum centrifuge at 50 °C for 10 min and covered with trypsin solution (Promega, 6.25 ng/ml in 50 mM ABC). Following rehydration with trypsin solution and removal of excess trypsin, the gel cubes were covered with 50 mM ABC and incubated overnight at 25 °C. Peptides were extracted from the gel cubes with 1% formic acid (FA) (once) and 5% FA/50% ACN (twice). All extracts were pooled and stored at −20 °C until use. Prior to liquid chromatography-mass spectrometry (LC-MS), the extracts were concentrated in a vacuum centrifuge at 60 °C, after which volumes were adjusted to 50 μl with 0.05% FA and filtered through a 0.45 μm spin filter into LC autosampler vials [13].

### NanoLC-MS/MS proteomic analysis and database searching

NanoLC-MS/MS analysis was performed as previously described [14]. In brief, peptides were separated using an Ultimate 3000 nanoLC system (Dionex LC-Packings, Amsterdam, The Netherlands) equipped with a 40 cm × 75 μm internal diameter (ID) fused silica column custom packed with 1.9 μm 120 Å ReproSil Pur C18 aqua (Dr Maisch GMBH, Ammerbuch-Entringen, Germany). The samples were injected by gel band, starting with gel band 1 at the top of the gel for all samples, followed by gel band 2, until the final gel band 5. The experiment was considered as one continuous injection series with a blank injection at the start of the experiment. After injection, peptides were trapped at 6 μl/min on a 1 cm × 100 μm ID trap column packed with 5 μm 120 Å ReproSil C18 aqua at 2% buffer B (buffer A: 0.05% formic acid in MQ; buffer B: 80% acetonitrile +0.05% formic acid in MQ) and separated at 300 nl/min in a 10–40% buffer B gradient for 75 min (100 min inject-to-inject). Eluting peptides were ionized at a potential of +2 kVa into a Q Exactive mass spectrometer (Thermo Fisher, Bremen, Germany). Intact masses were measured at resolution 70.000 (at m/z 200) in the Orbitrap using an AGC target value of 3E6 charges. The top 10 peptide signals (charge-states 2+ and higher) were submitted to MS/MS in the HCD (higher-energy collision) cell (1.6 amu isolation width, 25% normalized collision energy). MS/MS spectra were acquired at resolution 17.500 (at m/z 200) in the orbitrap using an AGC target value of 1E6 charges, a maxIT of 60 ms and an underfill ratio of 0.1%. Dynamic exclusion was applied with a repeat count of 1 and an exclusion time of 30 s. MS/MS spectra were searched against a Swissprot reference proteome FASTA file (release January 2018, 42,258 entries, canonical and isoforms, no fragments), using MaxQuant version 1.6.0.16 [15]. Enzyme specificity was set to trypsin and up to two missed cleavages were allowed. Cysteine carboxamidomethylation (Cys, +57.021464 Da) was treated as fixed modification and methionine oxidation (Met, +15.994915 Da) and N-terminal acetylation (N-terminal, +42.010565 Da) as variable modifications. Peptide precursor ions were searched with a maximum mass deviation of 4.5 ppm and fragment ions with a maximum mass deviation of 20 ppm. Peptide and protein identifications were filtered at a false discovery rate (FDR) of 1% using the decoy database strategy. Proteins that could not be differentiated based on MS/MS spectra alone were grouped to protein groups (default MaxQuant settings). A protein was considered identified when at least 1 unique peptide was identified in one sample at high confidence (peptide and protein FDR < 1%). Searches were performed with the label-free quantification option selected. Proteins detected were quantified based on MaxQuant (version 1.6.0.16) output data. Label-free quantification (LFQ) intensities were filtered by contaminants and only proteins with observations across all samples were retained. The MS proteomics data have been deposited to the ProteomeXchange Consortium via PRIDE [16] with accession number PXD015744.

### Weighted gene correlation network analysis (WGCNA) and functional enrichment of identified modules

A protein co-expression network is an undirected graph, where each node corresponds to a protein, and each edge connects a pair of proteins that are significantly correlated [17]. The key concept in WGCNA is “connectivity”. Connectivity describes direct and indirect relationships between two proteins/genes in networks [18].This metric has e.g. previously been used in breast cancer for drug prioritization by Neidlin et al. [19].

To investigate co-expressed proteins in resected patient PDAC samples, we used the WGCNA package [6] in R version 3.5.0 WGCNA defines modules as a group of densely interconnected proteins [18]. In unweighted networks the only information given is the correlation “yes” or “no”, while for weighted networks, users can also gain information about the strength of a correlation. To remove random noise and enhance the strength of correlation, a particular threshold is required from the user. In the WGCNA package and so in this study, the choice was made by applying the scale-free topology criterion [20] using a soft threshold also known as “beta power”. Different soft thresholds were tested (from 1 to 20) and power = 10 was retained to be enough to get an adjacency matrix very similar to a scale-free topology (correlation = 0.90) as shown in Supplementary Fig. S1. More explicitly, the adjacency matrix is obtained by using the correlation value between two proteins raised to the power threshold *β* (Formula 1)

$$ {a}_{(ij)}={s}_{(ij)}^{\beta } $$

where *a*_(ij)_ is the weighted value of a protein in the adjacency matrix defined by rising the co-expression similarity *s*_(ij)_ to a power *β*. Finally, modules are obtained by setting a cut-tree cut off on hierarchical clustering branches. In this study, a dynamic cut-tree method was chosen. Briefly, the algorithm starts by obtaining few large clusters by the static tree cut and, next, implements an iterative process of cluster decomposition and combination. The iteration stops only when the number of clusters becomes stable [21]. The dynamic tree method is essential to avoid relatively small modules. In this study, the minimum module size was set to 20 and the cut height was set to 0.998 automatically by the WGCNA tool. Each module was summarized by a vector of values (1 × 20 in this analysis, where 20 is the number of samples) that was called Module Eigenprotein (ME) and corresponded to the first principal component of the given module [22]. The final network was defined using the weighted option and threshold = 0.02 based on the value range of the data.

The GSEA of the modules was performed by mapping the proteins to gene names and submitting the gene list of each module to the GSEA Broad Institute browser [23]. Gene sets were ranked by significant *p* value and number of overrepresented genes. We adopted GSEA to perform functional enrichment analysis for each subnetwork based on GO biological process (BP) terms, cellular components (CC), hallmarks of cancer (HC) and transcription factor binding sites (TFBS). Each gene set was ranked using the FDR score and the number of overlapping genes between the module and the gene set. Moreover, the top 5 over-represented terms of each module were subjected to STRING analysis, in order to find the best descriptive biology for each specific module.

### Survival analysis and meta-analysis

Clinical data of patients undergoing resection were obtained from electronic patient records and referral hospitals. Survival data were obtained from government registration. The ME for each module was then correlated to DFS and OS. Assuming to have a trait *T* and a Module Eigenprotein (ME), correlation or univariate regression models can be used to measure the extent of their association. Modules with a high trait significance may underlie biological pathways associated with the sample trait. Meta-analysis was carried out by applying univariate Cox regression, multivariate Cox regression and log-rank tests on our proteomics dataset and on two different transcriptomics datasets (TCGA [24] and Moffitt et al. [25]). Prognostic marker candidates were ranked based on the number of significant *p* values obtained from the above-mentioned statistical tests. Kaplan-Meier curves were plotted using “survminer” package in R.

### Immunohistochemical validation of prognostic markers in an independent cohort

Immunohistochemistry (IHC) of tissue microarrays (TMAs) was evaluated as previously described [26]. In brief, FFPE tissues from resected patients were selected and combined in TMAs, including four representative cores from 4 different tumor areas for each patient. IHC staining of KHSRP, SPTBN1 and PYGL was performed according to the manufacturer’s protocols. Anti-KHSRP monoclonal antibody (1:200, anti-KHSRP rabbit ab150393 Abcam), anti-SPTBN1 monoclonal antibody (1:500, anti-SPTBN1 mouse MA3–062, Invitrogen) and anti-PYGL polyclonal antibody (1:150, anti-PYGL rabbit ab198268) were used. Visualization was obtained using a BenchMark Special Stain Automation system (Ventana Medical Systems, Export, USA). Protein staining was evaluated by a molecular pathologist, assessing the amount of tumor and tissue loss, background and overall interpretability. Cytoplasmic immunostaining intensity was classified into four grades: 0 (absent), 1 (weak), 2 (moderate) and 3 (strong), for both STPBN1 and PYGL. To reduce the scoring complexity, samples were defined as “with high expression”, when the staining score was >2 in at least 50% of the tumor cells. The nuclear immunostaining intensity of KHSRP was classified into two grades: 0 (absent) and 1 (present). All patients provided written informed consent for the storage and analysis of their tumor material and survival data, respectively. This study was approved by the Local Ethics Committee of the University of Pisa (Ethics approval #3909, July 3rd, 2013).

Univariate and multivariate analyses were performed using a Cox regression model. Proteins with HR (Hazard Ratio) < 1 were considered protective and those with HR > 1 were defined as non-protective. Meanwhile, proteins with *p* values < 0.05 were considered statistically significant. A risk score method was used to assess the association of the three prognostic markers with OS in a multivariate analysis. The risk score was evaluated by combining the TMA scores of prognostic proteins weighted by their regression coefficients from univariate Cox regression (Formula 2).

$$ Risk\ score=\sum \limits_{\mathrm{i}=1}^{\mathrm{n}}{\mathrm{TMA}}_{\mathrm{score}}\ast {\upbeta}_{\mathrm{i}.} $$

where n is the number of prognostic proteins, TMA_score_ is the score of TMA for protein i, and β_i_ the regression coefficient of protein i in the univariate Cox regression analysis.

Group comparisons were evaluated using the unpaired nonparametric Mann-Whitney U test or unpaired Student’s t test. Fishers exact test was used for categorical analysis. Correlations with clinicopathological characteristics, including DFS and OS, were tested using Kaplan-Meier curves and the log-rank test, as described above.

### Cell culture

Hs766t cells (ATCC, Manassas, USA) were grown in DMEM (Lonza, Verviers, Belgium) supplemented with 10% heat-inactivated fetal bovine serum and 1% penicillin-streptomycin (10,000 U/ml, Gibco, Gaithersburg, MD, USA). Cells were kept at 37 °C in an atmosphere of 5% CO_2_ in 75 cm^2^ tissue culture flasks (Greiner Bio-One GmbH, Frickenhausen, Germany) and, for all the experimental procedures, harvested using trypsin-EDTA (Sigma, Zwijndrecht, The Netherlands) in their exponentially growing phase. Cells were tested within the last 3 months by microscopic morphology check and growth curve analysis according to the Cell Line Verification Test Recommendations (ATCC-Technical Bulletin No. 8, 2008). Periodic assays were carried out to detect mycoplasma contamination, and the identity of the cells was confirmed by PCR profiling using short tandem repeats (STR).

### Immunofluorescence assay

Immunofluorescence analysis was performed according to a previously established protocol [27]. Briefly, cells were seeded in a Chamber-Slides System (Lab-Tek, Thermo Fisher Scientific, Waltham, USA) at a density of 5000 cells/well and allowed to attach overnight. Next, co-expression of KHSRP and SPTBN1 was evaluated in Hs766t PDAC cells, stained simultaneously with an anti-KHSRP monoclonal antibody (1:400, anti-KHSRP rabbit ab150393 Abcam) followed by an Alexa Flour 535 anti-rabbit antibody (Red; 1:70), and an anti-SPTBN1 monoclonal antibody (1:100, anti-SPTBN1 mouse MA3–062, Invitrogen) followed by an Alexa Flour 488 anti-mouse antibody (Green; 1:70). Nuclear DNA was stained with 4′, 6-diamidino-2-phenylindole (DAPI). Images were captured using a Zeiss Laser Scanning Microscope, processed and merged using Axiovision 4.1 software (Zeiss Microimaging, Thornwood, USA). In vitro experiments were performed with a minimum of three biological replicates, evaluating at least 100 cells.

## Results

### PDAC tissue proteomics and co-expression analysis

**Fig. 1:**
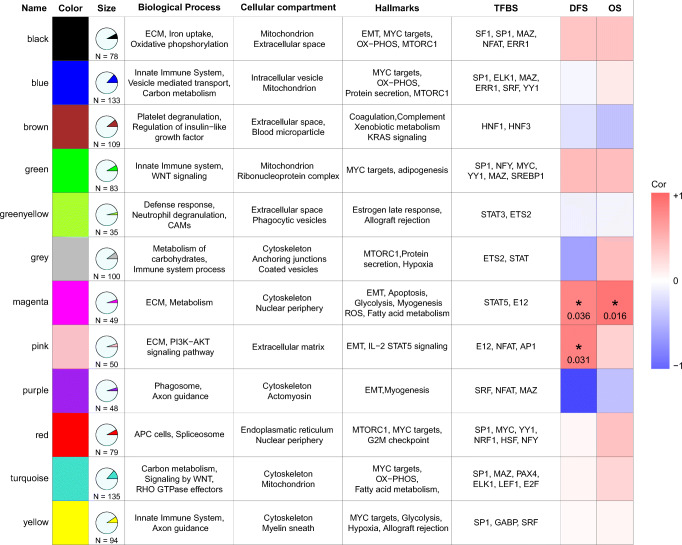
Descriptive table of module characteristics. Module names, colors and numbers of proteins are indicated. Enrichment of biological processes, cellular compartments and hallmarks of cancer are described for each module by GSEA. The last two columns show correlations and significance levels of clinical endpoints for each module. The magenta module shows a positive (red color) and significant correlation with DFS (*p* value 0.036) and OS (*p* value 0.016) while the pink module shows a positive and significant correlation with DFS (*p* value 0.031)

To obtain proteome level insight into PDAC cells, we used in-depth proteomics based on label-free nanoLC-MS/MS of gel-fractionated proteins to generate proteomic profiles of a cohort of 20 patients. The clinical characteristics of the selected patients are listed in Supplementary Table S1. We ensured equal protein loading of the samples to obtain optimal results (Supplementary Fig. S2). The obtained dataset consisted of 5667 proteins (contaminants removed) encoded by 5494 genes. Unsupervised clustering using all proteins did not reveal any specific grouping of the samples (Supplementary Fig. S3). The proteome dataset was subsequently used to establish a PDAC protein network. To obtain robust co-expression networks, we restricted the analysis to 993 proteins identified in all samples (Supplementary Table S2). Subsequent co-expression analysis yielded 12 consensus modules (Fig. 1), that were subsequently analyzed by GSEA to characterize the associated biology. Each module was annotated with gene sets and clinically relevant information. A complete list of genes associated with the modules is presented in Supplementary Table S3.

The modules covered a wide range of biological terms, and the most frequently occurring terms were those implicated in metabolic processes in context of the mitochondrial compartment (black, green, magenta, turquoise and yellow modules). Furthermore, five modules (blue, green, green yellow, grey and yellow) consisted predominantly of immune system and defense response, probably regulated by STAT3 and ETS2 transcription factors (shown in the TFBS column in Fig. 1), while one module (brown) was linked to coagulation and platelet activation. Four modules were associated with epithelial-to-mesenchymal transition (EMT) processes (black, magenta, pink and purple modules). One module was enriched for transcription factors with STAT5A and E12 binding sites. However, these binding sites were predicted based on the binding regions present in the targets. The transcription factors did not show over-expression in our PDAC cohort.

### Modules as potential prognostic markers for pancreatic cancer

**Fig. 2:**
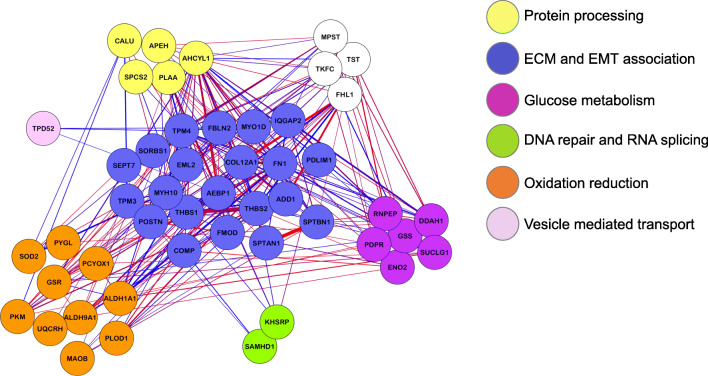
Network visualization of the magenta module that associates with DFS and OS. Protein names are mapped to genes through Uniprot. Edge’s widths represent the correlation strengths between genes (blue = negative correlation, red = positive correlation). Gene colors represent biological processes as indicated in the figure

The rationale behind the correlation network approach is to use the network language, which is particularly intuitive to biologists and allows for simple social network analogies. This method indeed allows the detection of biologically meaningful communities in the network and the study of relationships between them, helping the user to define interesting modules associated to external traits. Since co-expressed protein modules were identified and associated with hallmarks of cancer, we hypothesized that some modules may harbor potential markers for PDAC prognosis. Indeed, the magenta module, which presents EMT and glycolysis pathway components, exhibited positive and significant correlations with DFS and OS in our cohort (Fig. 1). Subsequently, we explored the network biology of the prognostic co-expression module and found that this module is involved in metabolism, as can be inferred from the presence of PYGL, SOD2, GSR, GSS, PKM2, DDAH1 and TST, as well as EMT through ENO2, PLOD1 and FMOD (Fig. 2). Moreover, factors exclusively related to EMT were: COL12A1, TPM4, THBS1, FN1, POSTN, COMP, THBS2, CALU and FBLN2 (Fig. 2).

### The Magenta module comprises candidate prognostic biomarkers for resected PDAC

**Fig. 3:**
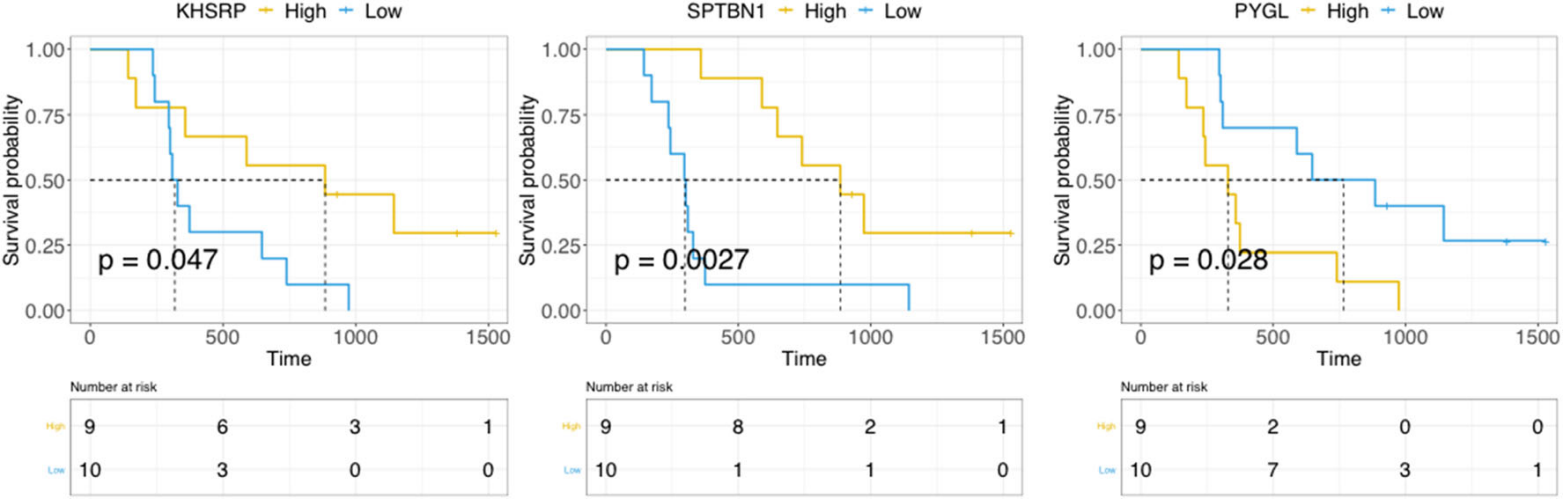
Candidate biomarkers deduced from PDAC proteomics data. Kaplan-Meier curves of KHSRP, SPTBN1, KHSRP and PYGL proteomics data

For each module, we obtained the Module Eigenprotein (ME) for further survival association analyses. Only one module was significantly associated with DFS and OS in our proteomics cohort. The same protein signature was tested for OS association with transcriptomics data of the TCGA-PAAD project. The *p* value for all overall tests (i.e., Likelihood, Wald and Log Rank score) were 0.007, 0.01 and 0.01, respectively, indicating that the same gene signature is significantly associated to OS also in the transcriptomics data. Through subsequent investigation of epigenetic alterations of those genes, we found that oxidative stress and ECM-EMT related genes were not methylated. Therefore, we conclude that epigenetic inhibition of these genes was not prevalent. Additionally, we explored whether it was possible to refine the prognostic signature list by analyzing two publicly available independent transcriptomics datasets [24, 25]. To this end, different statistical tests were applied to prioritize the prognostic candidates, and the genes were ranked based on the frequency of significant observations among the tests (Supplementary Table S4). Our analysis revealed three potential top candidate biomarkers linked with prognosis: scaffold membrane protein spectrin beta chain, non-erythrocytic-1 (SPTBN1), splicing regulatory protein KHSRP and glycogen phosphorylase (PYGL) (Fig. 3). SPTBN1 is an actin-crosslinking protein that links the plasma membrane to the actin cytoskeleton. KHSRP is a multifunctional RNA-binding protein implicated in transcription, pre-mRNA splicing and mRNA localization to control important cellular processes such as metabolism, immune response, proliferation and differentiation. PYGL is a crucial phosphorylase that catalyzes the release of glucose molecules from glycogen, the major carbohydrate storage source. Cells under hypoxic conditions accelerate glycogen metabolism for an optimal glucose utilization (Warburg effect). Thus, PYGL is required for hypoxic cancer cells (as pancreatic cancer cells typically are) for glycolysis and glycogen degradation [28]. Based on genetic data from the TCGA consortium we found that alterations on PYGL can discriminate patient survival (*p* value < 0.001) even though the number of samples for short survival was relatively small. Interestingly, we found that high expression of SPTBN1 was associated with good prognosis in the proteomics data, but with poor prognosis in the transcriptomics data (Supplementary Fig. S4). Correlations between mRNA and protein data have been extensively studied and debated in the past years [29–31] and includes two recent large-scale clinical cancer proteo-genomics studies. A more recent study by Vasaikar and colleagues [32] showed that enzymes belonging to the tricarboxylic acid (TCA) may be universally decreased at the protein level, but not at the mRNA level. This suggests a protein-level adaptation driving a strong Warburg effect in microsatellite instable (MSI) colorectal cancer. In agreement with our study, the module where SPTBN1 belongs to is strongly enriched for metabolic genes. More specifically, these genes belong to glycolytic effects (PYGL, PKM, ENO2), thus preceding the TCA cycle. Another study by Mertins and colleagues [33] on breast cancer showed that despite a C-terminal truncation of GATA3, its protein expression level did not decrease, suggesting the occurrence of post-translational modification. Furthermore, these researchers found that signaling pathways such as PS1, ion channel transport and proteasome and basic cellular mechanism pathways, including ribosome, mRNA splicing, glycosylphosphatidylinositol biosynthesis and RNA polymerase, were enriched for negative correlations between mRNA and protein levels when compared to copy number alterations. Overall these findings suggest that post-translation modifications are more prone to occur in specific pathways compared to others. Since SPTBN1 has also been shown to carry genetic alterations in hepatocellular carcinoma patients with a short OS [34], this may be a starting point for future investigations on SPTBN1 mutations in PDAC patients.

### Validation of KHSRP, SPTBN1 and PYGL as prognostic candidates for resected PDAC

**Fig. 4:**
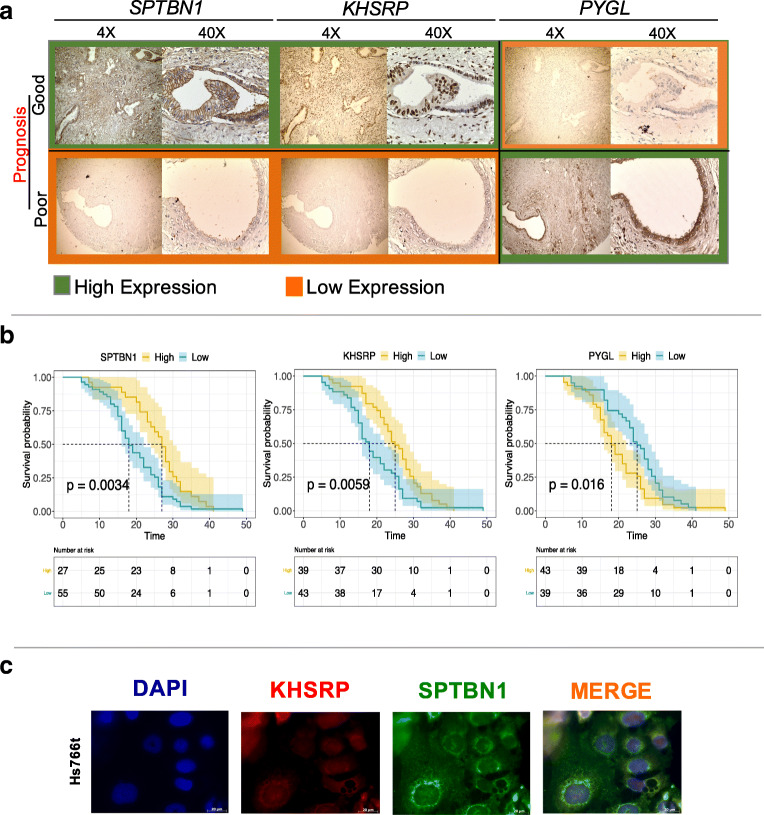
SPTBN1, KHSRP and PYGL as prognostic markers for resected pancreatic cancer patients. **a**. Immunohistochemistry validation of SPTBN1, KHSRP and PYGL on TMAs of 82 patients. **b**. Kaplan-Meier curves for SPTBN1, KHSRP and PYGL with *p* values 0.0034, 0.0059 and 0.016, respectively. **c**. Immunofluorescence of KHSRP (red) and SPTBN1 (green) in Hs766t cells

We used WGCNA with unsigned networks. This means that the proteins in our modules can show both positive or negative correlations and that poor or good prognostic markers can fall in the same module because they are associated with the same biology. The top 3 prognostic markers, SPTBN1, KHSRP and PYGL, were chosen for subsequent IHC validation in an independent cohort of 82 resected PDAC patients (Fig. 4). Representative IHC images of tumor cores from two selected patients with highly divergent survival times and their SPTBN1, KHSRP and PYGL expression patterns are shown in Fig. 4a. In line with the proteome data, we found that SPTBN1 and KHSRP correlated with each other and were overexpressed in patients with good prognosis while PYGL, that anti-correlates with KHSRP and SPTBN1, correlated with poor prognosis. All three proteins had a significant prognostic value for OS (Fig. 4b) and PFS (Supplementary Fig. S5). Moreover, the signature of the three proteins taken together successfully predicted patient prognosis with *p =* 0.0025 (Supplementary Fig. S6). Co-expression of SPTBN1 and KHSRP was further confirmed by immunofluorescence in Hs766t cells. KHSRP (nuclear protein) and SPTBN1 (cytoplasmic protein) were clearly co-expressed (Fig. 4d) in the nucleus and in the cytoplasmatic compartment, respectively.

Finally, univariate and multivariate Cox regression models were used to assess the association of the three prognostic markers to OS. We found that SPTBN1, KHSRP and PYGL maintained significance in univariate and multivariate analyses when correcting for external factors. To assess whether all the three proteins were significantly associated with OS in a multivariate analysis, a risk score was evaluated showing that the prognostic signature of these three proteins was highly associated with OS in this independent cohort (Table 2).

**Table 2:**
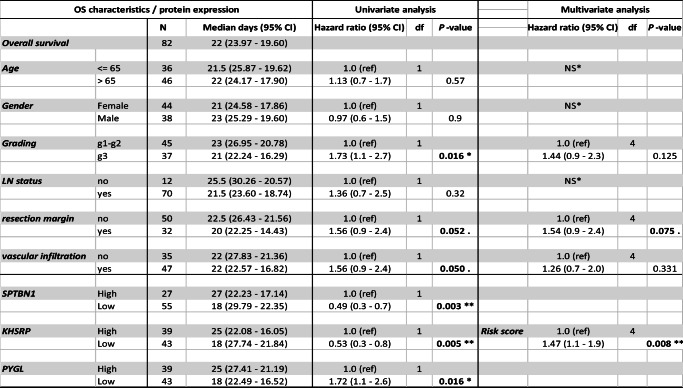
Univariate and multivariate analysis of prognostic markers for resected PDAC

Validation cohort characteristics with univariate and multivariate analyses for factors associated with OS. SPTBN1, KHSRP and PYGL remain significantly associated with OS together with grading stage, resection margin and vascular infiltration (significant *p* value in bold). In the multivariate analysis, significant covariates from univariate analysis are included and SPTBN1, KHSRP and PYGL are combined under the risk score

*NS: not significant in univariate cox regression; CI: Confidence of interval; df: degree of freedom

## Discussion

In the present study, we generated a comprehensive proteome dataset of 20 resected PDAC specimens and applied a weighted gene co-expression network analysis (WGCNA) to the data. WGCNA is a user-friendly and comprehensive software tool that has already been applied to several clinical features including brain cancer [35], diabetes [36] and chronic fatigue [37]. We focused on co-expression network analysis to infer biological functions and novel prognostic PDAC biomarkers. We reported a proteome dataset of 5667 proteins comprising 993 proteins identified in all samples giving rise to twelve modules in total. Protein co-expression modules were linked to well-known PDAC hallmarks of cancer such as axon-guidance, EMT, oxidative phosphorylation, MYC targets and KRAS signalling, as well as potential new relationships to biological processes. Importantly, one module was found to be significantly associated with survival. This module, called “magenta”, was functionally enriched for glycolysis, EMT, apoptosis and reactive oxidative stress, highlighting a possible interplay between these biological processes.

Despite considerable experimental and computational modeling efforts, the role of EMT in cancer is still not fully understood [38]. In particular, the connection of EMT to various properties of cancer cells such as stemness, drug resistance, metabolism and metastasis is heavily discussed [39, 40]. In our current study, four modules showed overrepresentation of different sets of EMT genes (black, magenta, pink, purple), correlating with metabolic pathways, suggesting that cell metabolism can influence the EMT state or vice versa. Previously, tumor metabolism has been found to be associated with EMT [40, 41], illustrating the complexity of the interplay between EMT and metabolic reprogramming. Interestingly, these four modules were regulated by different transcription factors. In the magenta module transcription factors binding sites (TFBS) for STAT5 and E12 were noted. STAT5 has been shown to be overexpressed during EMT and aberrant activity of this transcription factor has been found to induce mitochondrial dysfunction and reactive oxygen species (ROS) formation, leading to DNA damage [42]. In addition, E12 has been found to be associated with repression of E-cadherin (and thus EMT) in mouse models [43].

Of note, all modules with SP1 as transcription factor binding site (yellow, turquoise, red, green, blue, black) where found to be associated with MYC targets as previously described [44, 45]. SP1 has been shown to regulate the expression of thousands of genes implicated in the control of a diverse array of cellular processes, such as growth [44], differentiation [46], apoptosis [44], angiogenesis [47] and immune response [48]. These cellular processes are all linked to the proteomic modules of our cohort that present SP1 as putative transcription factor.

The magenta module comprised three prognostic markers: SPTBN1, KHSRP and PYGL that were subsequently validated in an independent cohort of 82 patients. These markers may be used in the future to evaluate and predict clinical responses of PDAC resected patients. SPTBN1 is a dynamic intracellular non-pleckstrin homology-domain protein, which plays important roles in cellular shape formation, protection of membranes against stress, positioning of transmembrane proteins, and molecular trafficking. Spectrin is made up of four subunits. Among these, the beta subunits are responsible for most of the binding activity and its role as a transforming growth factor-β signal transducing adapter protein that is necessary to form Smad3/Smad4 complexes [49]. KHSRP (KH-Type Splicing Regulatory Protein) controls important cellular processes such as proliferation, differentiation and metabolism. KHSRP (also known as FBP2) is a factor interacting with an enhancer element upstream of the *c-MYC* oncogene promoter [50]. In the past twenty years additional roles of KHSRP in post-transcriptional control of gene expression have been discovered with implications for pre-mRNA splicing [51], mRNA decay [52] and microRNA biogenesis [53]. PYGL catalyzes the degradation of glycogen [54] and is responsible for maintaining blood glucose homeostasis by regulating the release of glucose 1-phosphate from liver glycogen stores [55].

Importantly, transcriptomics data of all three biomarkers revealed significant associations with survival. Interestingly, SPTBN1 could be defined as a good prognostic marker based on the proteomics as well as the protein-based IHC data, while it was associated with poor prognosis based on the transcriptomics data (Supplementary Fig. S4A). Systematic studies have revealed multiple processes beyond the “non-correlation” of mRNA expression and protein concentration levels [56]. These include (i) specific translation rates of e.g. upstream open reading frames (uORFs) [57] or internal ribosome entry sites (IRES), (ii) translation rate modulation due to the binding of regulatory proteins or binding of micro-RNAs [58], (iii) modulation of a protein half-life involving the complex ubiquitin-proteasome pathway [59], or autophagy, which may influence protein concentrations independent of transcript levels.

Although we captured three new prognostic biomarkers and the biology associated with these, there are some limitations to our study that need to be noted. Due to the high heterogeneity of PDAC and the limited number of samples, we were not able to delineate proteomics-based PDAC subtypes. Exploring correspondence or correlation with known PDAC subtypes is challenging due to the lack of PDAC subtypes based on proteomics data. Furthermore, because of the limited number of samples, this study should be considered as a first exploratory analysis and its prognostic relevance needs to be validated in additional clinical studies.

Taking together, our data indicate that an EMT-metabolic module is associated with the prognosis after surgical resection of PDAC patients and that the module’s proteins SPTBN1, KHSRP and PYGL may serve as potential prognostic biomarkers. Our results also show that co-expression networks are able to extrapolate tumor-specific biology as well as biological mechanisms empowering prognostic marker discovery, even with a limited number of samples.

## Electronic supplementary material

ESM 1 (DOCX 60 kb)

## Abbreviations

WGCNA: weighted gene co-expression network analysis
PDAC: Pancreatic ductal adenocarcinoma
ME: Module Eigenprotein
EMT: Epithelial-mesenchymal-transition
OX-PHOS: Oxidative phosphorylation
TFBS: Transcription factor binding site
OS: Overall survival
DFS: Disease-free survival
