# Supplementary material for: Co-expression analysis of pancreatic cancer proteome reveals biology and prognostic biomarkers
Source: Cell Oncol (Dordr). 2020 Aug 29;43(6):1147–59. doi: 10.1007/s13402-020-00548-y (PMC7716908; doi:10.1007/s13402-020-00548-y)
Supplement: Supplementary file 1 — (DOCX 60 kb) [file 13402_2020_548_MOESM1_ESM.docx]

Link to supplementary data <https://www.dropbox.com/sh/syh2lcj048b8mwy/AABw8cmxz8wxQ10oA6l0sbIqa?dl=0>

- TableS1: Clinicopathological data
- TableS2: Protein expression data
- TableS3: Modules Genes
- TableS4: Candidate biomarkers
- FigureS1: Soft threshold WGCNA
- FigureS2: Gel images
- FigureS3: Unsupervised clustering
- FigureS4: KM curves for KHSRP, SPTBN1 and PYGL on TCGA data.
- Figure S5: KM curves of KHSRP, SPTBN1 and PYGL on validation cohort’s PFS data.
- Figure S6: KM curve of KHSRP, SPTBN1 and PYGL taken together. Group 1 define patients with high expression of SPTBN1 and KHSRP, and low expression of PYGL; Group2 define the opposite, so low values of SPTBN1 and KHSRP, and high score for PYGL.
